# Incision Decision: Vertical or Horizontal Laparotomy in Traumatically Injured Pregnant Patients

**DOI:** 10.7759/cureus.115897

**Published:** 2026-09-07

**Authors:** Lucy Hart, Marissa Platner, Katherine Russo, Courtney H Meyer, Zachary Grady, Adora Santos, Helen Oleary, Janelle Tanghal, Christine Castater, Randi N Smith, Jason D Sciarretta, Jane Ellis, Jonathan Nguyen

**Affiliations:** 1 Department of Surgery, Emory University School of Medicine, Atlanta, USA; 2 Department of Acute Care Surgery, Grady Health System, Atlanta, USA; 3 Department of Obstetrics and Gynecology, Grady Health System, Atlanta, USA; 4 Department of Obstetrics and Gynecology, Emory University School of Medicine, Atlanta, USA; 5 Department of Surgery, Morehouse School of Medicine, Atlanta, USA

**Keywords:** celiotomy incision, laparotomy incision, maternal mortality or death, obstetric trauma, trauma in pregnancy

## Abstract

Background

Trauma is the leading cause of non-obstetric mortality in pregnant patients. While guidelines exist for the overall management of pregnant trauma patients, there is currently no consensus regarding the optimal laparotomy incision type in those requiring urgent operative intervention.

Objective

The purpose of this study is to compare the presentation, operative management, and outcomes between pregnant trauma patients who underwent a vertical versus a horizontal laparotomy incision for urgent operative intervention.

Study design

A retrospective cohort review was conducted at an American College of Surgeons-verified Level I trauma center from 2016 to 2022, including all pregnant trauma patients. For those requiring urgent operative interventions, outcomes were compared between patients who had a vertical versus a horizontal laparotomy incision.

Results

A total of 147 pregnant trauma patients were identified. Of those, 30 (20.4%) required urgent operative intervention and met inclusion criteria, with 11 (36.7%) patients in the midline laparotomy cohort and 19 (63.3%) in the horizontal incision cohort. Patients were similar in age, presenting heart rate, presenting GCS, Injury Severity Score (ISS), and hospital length of stay. Patients who ultimately required a midline laparotomy were more likely to be hypotensive on presentation (systolic blood pressure (SBP) of 86 vs. 120 mmHg, p = 0.005) and were more likely to be operatively co-managed by both trauma and obstetrics. Time from incision to delivery was similar for both vertical and horizontal incisions. Mortality in the vertical incision group was three (27.3%) patients versus zero (0.0%) in the horizontal incision group (p = 0.041).

Conclusion

This single-institution cohort is the first descriptive evaluation of laparotomy incision type in operative obstetric trauma. In the absence of signs of concomitant intra-abdominal maternal injury, a horizontal incision can be considered for select pregnant trauma patients with non-reassuring fetal heart patterns. Conversely, patients with severe maternal polytrauma or hypotension should be considered for a vertical incision. Together, these findings support current practice patterns.

## Introduction

Traumatic injury is a leading cause of maternal mortality during pregnancy and affects an estimated 5%-8% of all pregnant patients [1-3]. Management of obstetric trauma can be complex, as the needs of both the mother and fetus must be considered. A multidisciplinary team of obstetricians, trauma surgeons, neonatologists, and emergency department personnel is essential to optimizing care of both mother and fetus [4].

Indications for operative intervention among obstetric trauma patients vary significantly, ranging from non-reassuring fetal heart patterns to hemorrhagic shock and polytrauma. These varying presentations require different operative approaches. In terms of laparotomy incision, the general practice is that delivery for fetal indications in a stable mother may be managed with a horizontal incision, while a vertical laparotomy incision is indicated in the presence of maternal instability or suspected abdominopelvic injury.

In the emergent cesarean delivery literature, vertical incisions shortened median incision-to-delivery times, without an associated improvement in neonatal outcomes [5]. For an urgent, time-sensitive delivery, a vertical incision offers expedited access to the uterus and decreased risk of bleeding from the inferior epigastric arteries, and it is the preferred approach in perimortem cesarean delivery [6]. However, horizontal incisions continue to be frequently utilized by obstetricians, accounting for over 70% of emergency cesarean deliveries [5].

Currently, there are no consensus guidelines on the indications for a horizontal versus vertical laparotomy incision in the surgical management of obstetric trauma. Furthermore, data specific to incision selection in obstetric trauma are lacking, limiting the evidence supporting current practice or informing guideline development. The purpose of this study is to compare surgical management and outcomes among pregnant trauma patients who require operative intervention. Specifically, we compare demographics, presentation, management, and outcomes among patients who receive vertical or horizontal laparotomy incisions.

## Materials and methods

A retrospective cohort study was conducted at a high-volume academic Level I trauma center. The study was approved by the institutional review board (IRB#1180641). The institutional trauma registry was queried for all pregnant trauma patients who presented between January 1, 2016, and January 1, 2022. Pregnancy status was confirmed through chart reviews. Patients were eligible for inclusion if they required urgent abdominal operative intervention, defined as an abdominal operation within the first 24 hours of arrival. Patients were excluded if they were managed nonoperatively or underwent abdominal operation beyond 24 hours from arrival. The included population was then stratified based on the laparotomy incision used for abdominal surgical management: horizontal versus vertical incision.

Demographics, injury mechanism, Injury Severity Score (ISS), presenting vital signs, and outcomes were obtained from the trauma registry. Gestational age, operative indication and intervention, incision type, operative team, and timing of operative intervention and delivery were abstracted through manual chart review. Skin incision type was determined from the operative report, and operative indication was classified according to the documented indication in the operative note. Time of skin incision and neonatal delivery were determined from intraoperative records and used to calculate incision-to-delivery time. Primary outcomes included time from incision to delivery and need to convert to another incision. Secondary outcomes included hospital length of stay, maternal mortality, and fetal demise.

The above variables were compared between the two groups using univariate analysis. Categorical variables were presented as frequencies and percentages and compared using Fisher’s exact test for variables with cell counts <5, or Chi-square analysis for variables with cell counts ≥5. Continuous variables were expressed as medians with interquartile ranges (IQR), and then compared using the Median Test. All statistical analysis was performed using IBM SPSS Statistics for Windows, Version 29.0 (Released 2022; IBM Corp., Armonk, NY, USA).

## Results

During the study period, a total of 147 obstetric trauma patients were identified; 30 (20.4%) required urgent operative intervention and met the inclusion criteria for this study. Of those operative obstetric trauma patients, 11 (36.7%) underwent vertical laparotomy incisions, while 19 (63.3%) underwent horizontal laparotomy incisions.

Both vertical and horizontal incision cohorts were demographically similar. The two groups were similar in age, race, and injury mechanism, with the most common being motor vehicle collisions. There was no statistically significant difference in median ISS, gestational age, heart rate, or Glasgow Coma Scale (GCS). Those who required vertical incisions presented with a lower systolic blood pressure (SBP; 86 vs. 120 mmHg, p = 0.005). Those who underwent horizontal incisions were more likely to be 24 weeks of gestation or greater (100.0% vs. 63.6%, p = 0.005) (Table 1).

**Table 1:** Demographics and clinical presentation among pregnant trauma patients requiring emergent surgical intervention, stratified by incision type MVC: motor vehicle collision, GCS: Glasgow Coma Scale, SBP: systolic blood pressure, HR: heart rate, ISS: Injury Severity Score, MTP: massive transfusion protocol, ICU: intensive care unit.

Variable	Vertical incision (n = 11)	Horizontal incision (n = 19)	p-value
Age, years, median (IQR)	26 (18-34)	25 (21-31)	1.000
Race, n (%)			
Black	8 (72.7%)	15 (78.9%)	0.371
White	1 (9.1%)	1 (5.3%)	
Asian	0 (0.0%)	2 (10.5%)	
Unknown	2 (18.2%)	1 (5.3%)	
Injury type (blunt), n (%)	9 (81.8%)	18 (94.7%)	0.256
Mechanism of injury, n (%)			
MVC	8 (72.7%)	13 (68.4	0.402
Penetrating	2 (18.2%)	1 (5.3%)	
Assault	0 (0.0%)	3 (15.8%)	
Pedestrian vs. auto	1 (9.1%)	2 (10.5%)	
GCS, median (IQR)	14.5 (9-15)	15 (14-15)	0.083
SBP (mmHg), median (IQR)	86 (76-110.5)	120 (105-132)	0.005*
HR (beats per minute), median (IQR)	114.5 (85.5-146.25)	106 (92-115)	0.450
ISS, median (IQR)	26 (19-45)	17 (9-27)	0.450
MTP activation, n (%)	4 (36.4%)	2 (10.5%)	0.160
Gestational age (weeks), median (IQR)	30 (18-36)	34 (30-35)	0.450
Gestational age ≥24 weeks, n (%)	7 (63.6%)	19 (100.0%)	0.005*

Indication for operative intervention differed significantly between the two groups. For those who received vertical incisions, the most frequent indication was non-reassuring fetal heart tones (NRFHTs) in five patients (45.5%), followed by hemoperitoneum in four (36.4%), both in the context of maternal hypotension. For those who had a horizontal incision, NRFHT was also the most common indication, occurring in 17 patients (89.5%), followed by suspected placental abruption in 2 (10.5%), with hemoperitoneum never identified as the operative indication in this group (p = 0.004).

Multidisciplinary operative management differed for patients depending on whether they underwent a vertical or horizontal incision. The operative team for patients undergoing vertical laparotomy incisions frequently included both trauma surgery and obstetrics in eight patients (72.7%), followed by trauma surgery alone in two (18.2%), and, lastly, obstetrics alone in one (9.1%). Conversely, those undergoing horizontal incisions overwhelmingly underwent operative intervention with the obstetrics team alone in 16 patients (84.2%) and less commonly by a collaborative trauma and obstetrics team in 3 (15.8%). No patients in the horizontal incision group were operatively managed by the trauma team alone (p < 0.001) (Table 2). The two patients in the vertical incision group managed operatively by the trauma team alone underwent exploratory laparotomy without cesarean delivery and did not contribute to the incision-to-delivery analysis. All cesarean deliveries across both groups were performed by the obstetrics team, including an OB attending.

**Table 2:** Multidisciplinary operative management of obstetric trauma

Variable	Vertical incision (n = 11)	Horizontal incision (n = 19)	p-value
Operative team, n (%)			
OB/GYN	1 (9.1%)	16 (84.2%)	<0.001*
Trauma surgery	2 (18.2%)	0 (0.0%)	
Both trauma and OB/GYN	8 (72.7%)	3 (15.8%)	
Indication for operative intervention, n (%)			
Suspected placental abruption	0 (0.0%)	2 (10.5%)	0.004*
Intrauterine fetal demise	2 (18.2%)	0 (0.0%)	
Non-reassuring fetal heart tones	5 (45.5%)	17 (89.5%)	
Hemoperitoneum	4 (36.4%)	0 (0.0%)	
Time to delivery (minutes), median (IQR)			
From ED arrival to delivery	62 (18.5-128.5)	117 (77-438)	0.103
From incision to delivery	2 (1.25-3.75)	3 (1-7)	0.678

Incision-to-delivery time was assessed among the 28 patients who underwent cesarean delivery during the index operation, including 9 vertical patients and 19 horizontal patients. There was no significant difference in time from incision to delivery between the two incision types (two vs. three minutes, p = 0.678), though the horizontal incision group demonstrated a wider range and spread of incision-to-delivery times (Figure 1). The horizontal incision group had a longer median time from ED arrival to delivery when compared to the vertical incision group, though this was not statistically significant (62 vs. 117 minutes, p = 0.103).

**Figure 1:**
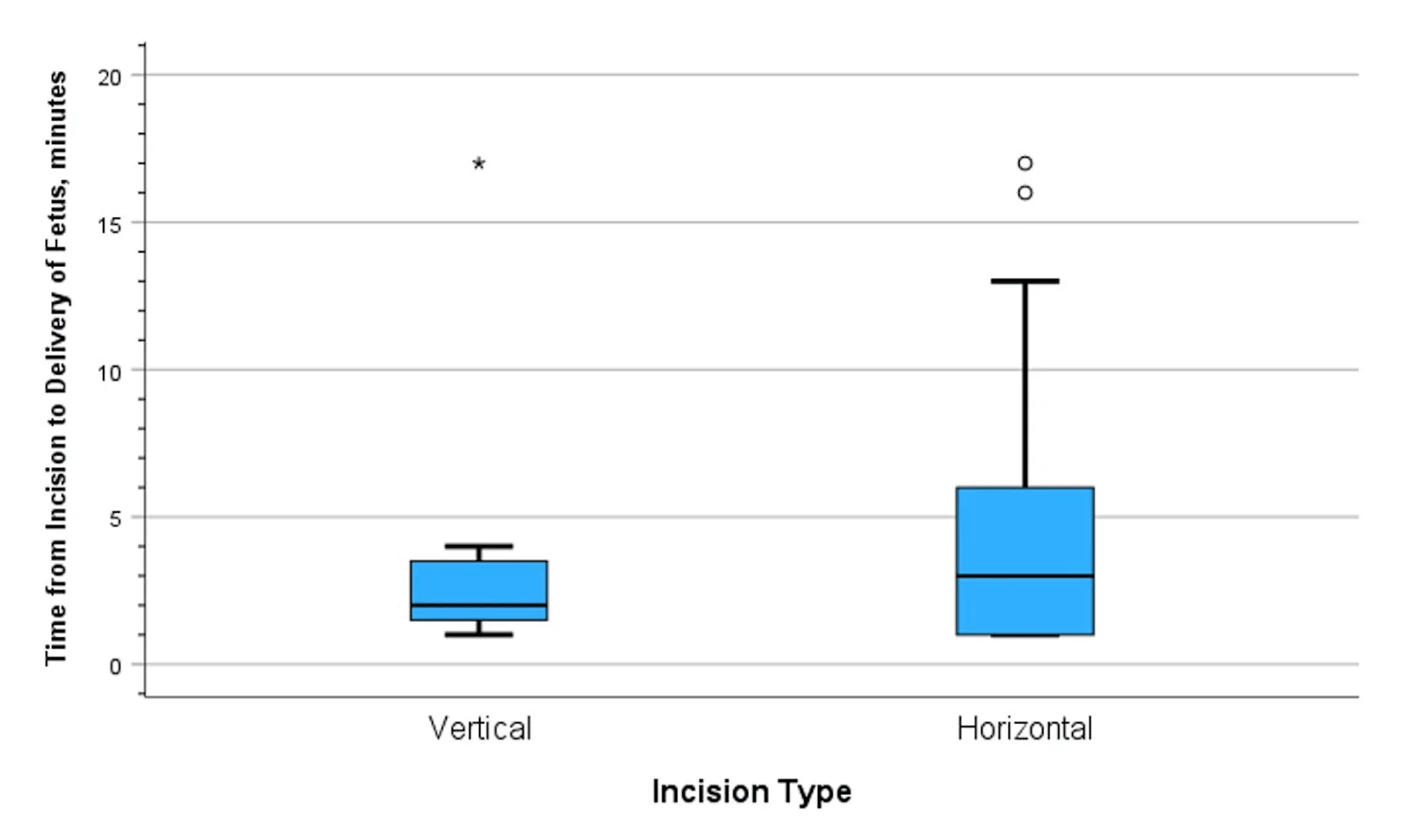
Box plot of incision-to-delivery times, stratified by incision type

The presence of concomitant injuries differed between the vertical and horizontal incision groups. Those who received vertical incisions were more likely to have hollow viscus injuries (27.3% vs. 0.0%, p = 0.041), solid organ injuries (45.5% vs. 0.0%, p = 0.003), and uterine rupture (36.4% vs. 0.0%, p = 0.012). The vertical incision cohort had higher rates overall of concomitant abdominopelvic trauma (81.8% vs. 0.0%, p < 0.001) when compared to the horizontal incision cohort. Those who underwent a horizontal incision had a higher incidence of placental abruption (84.2% vs. 45.5%, p = 0.026). Maternal and fetal outcomes differed significantly between groups. The vertical incision group had higher rates of both maternal mortality (27.3% vs. 0.0%, p = 0.041) and fetal demise (63.6% vs. 5.3%, p = 0.001) (Table 3). No patient in the horizontal incision group required conversion to a vertical incision.

**Table 3:** Findings, concomitant injuries, and outcomes

Variable	Vertical incision (n = 11)	Horizontal incision (n = 19)	p-value
Presence of concomitant abdominal or pelvic trauma, n (%)	9 (81.8%)	0 (0.0%)	0.001*
Hollow viscus injury	3 (27.3%)	0 (0.0%)	0.041*
Solid organ injury (spleen, kidney, liver)	5 (45.5%)	0 (0.0%)	0.003*
Abdominopelvic vascular injury	2 (18.2%)	0 (0.0%)	0.126
Uterine rupture	4 (36.4%)	0 (0.0%)	0.012*
Placental abruption, n (%)	5 (45.5%)	16 (84.2%)	0.026*
Total hospital days, median (IQR)	5 (3-12)	7.5 (3-13)	0.695
Maternal mortality, n (%)	3 (27.3%)	0 (0.0%)	0.041*
Fetal mortality, n (%)	7 (63.6%)	1 (5.3%)	0.001*

## Discussion

In this study, we compared vertical and horizontal abdominal incisions in the operative management of pregnant trauma patients. Vertical incisions were performed predominantly in patients with hemodynamic instability or suspected concomitant abdominopelvic trauma. Incision-to-delivery times did not differ significantly between groups, though these findings should be interpreted with caution given the sample size. Patients undergoing a vertical incision had higher maternal and fetal mortality than those operated on for isolated fetal indications, likely reflecting injury severity rather than incision selection.

The decision to approach the abdomen through a vertical versus horizontal incision can be critical in ensuring the safety of the mother while balancing the need for expedient delivery of the fetus. Conventional teaching holds that the presence of concomitant abdominopelvic trauma or maternal instability necessitates the use of a vertical incision to allow for adequate abdominal exploration and control of life-threatening maternal injuries, while delivery for isolated fetal indications may be performed through a horizontal incision [5,7]. Although broadly adopted in the management of obstetric trauma, the literature supporting this practice remains limited.

Our institution follows this practice, and obstetric trauma patients with hemodynamic instability or suspected concomitant injuries are generally managed with an emergent vertical laparotomy. Our findings reflect this, as patients who underwent a vertical incision presented with lower SBP and had significantly higher rates of concomitant abdominopelvic injury, including solid organ injury, hollow viscus injury, and uterine rupture. No patients in the horizontal incision group had concomitant abdominopelvic injuries, and none required conversion to a vertical incision, suggesting that preoperative assessment reliably identified patients without maternal intra-abdominal injury in this cohort.

Expedient delivery is integral in ensuring both maternal and fetal safety, and a vertical incision has historically been suggested to allow faster access to the uterus [5]. In our small cohort, time from incision to delivery did not differ significantly between incision types. Our data are consistent with a practice in which vertical incisions are selected for exposure of potential maternal injury rather than for expedited delivery alone. Incision selection also involves considerations beyond speed of delivery, as the horizontal incision carries increased risk of hemorrhage from injury to the inferior epigastric vessels, particularly in the setting of coagulopathy [6]. Along with the concern for limited exposure for rapid control of injuries to the upper abdomen and retroperitoneum, these considerations further support reserving the horizontal incision for patients without suspected maternal intra-abdominal injury.

A multidisciplinary team of anesthesiologists, obstetricians, neonatologists, and trauma surgeons is integral in caring for obstetric trauma patients [4]. In this study, patients undergoing horizontal incisions for isolated fetal indications were more frequently managed operatively by the obstetrics team alone. Conversely, patients undergoing vertical incisions with concern for concomitant injury were usually operatively managed by both trauma and obstetric teams. All obstetric trauma patients were evaluated and co-managed by both services outside of the operating room. At our institution, incision selection is determined by the operating attending surgeon, reflecting a multidisciplinary discussion between trauma surgery and obstetrics when both services are present.

Outcomes among operative obstetric trauma patients remain poor, particularly among those with concomitant abdominopelvic trauma [1,7]. In this study, maternal mortality and fetal demise were higher in the vertical incision group. This difference likely reflects injury severity rather than incision type, and it is consistent with prior literature demonstrating worse outcomes among obstetric trauma patients with concomitant shock or abdominopelvic injuries.

Clinical implications

Obstetric trauma is a significant contributor to maternal morbidity and mortality, and optimal management requires a multidisciplinary approach, particularly in complex operative intra-abdominal trauma. While the decision to perform a cesarean delivery depends on maternal and fetal status, the choice of incision type (vertical or horizontal) has historically varied with the specific patient presentation. In our cohort, delivery was accomplished through either incision type without a statistically significant difference in incision-to-delivery time. These findings are descriptively consistent with the long-held surgical principle that a vertical incision is indicated when maternal instability or intra-abdominal injury is suspected, while a horizontal incision may be reasonable for patients with isolated fetal indications and no evidence of maternal abdominopelvic injury.

Strengths and limitations

This study presents primary data on a clinical scenario for which published evidence is limited. The trauma volume at this center permits assembly of a cohort in a rare clinical setting, and detailed manual chart review yielded granular data on operative indication and timing for each patient.

Ultimately, there are limitations to this study. The small sample size limits statistical power, and results should be interpreted cautiously. The retrospective, single-center design precludes causal inference and limits generalizability. The specific team member performing each portion of the case was not consistently documented in the operative record and may confound the incision-to-delivery time comparison. The rationale for incision selection was similarly not documented and could not be reliably abstracted. The degree of fetal distress in patients operated on for fetal indications was not quantified, and long-term maternal and neonatal outcomes were not available for this study. Multicenter analysis would improve cohort size and generalizability, and further evaluation of the adequacy of abdominal exploration through a horizontal incision and the concern for associated risk of hemorrhage in this population is warranted.

## Conclusions

This single-institution cohort provides the first descriptive evaluation of laparotomy incision type in the operative management of obstetric trauma. In the absence of signs of concomitant intra-abdominal maternal injury, a horizontal incision can be considered for select pregnant trauma patients with non-reassuring fetal heart patterns. In contrast, patients with severe maternal polytrauma or hypotension should be considered for a vertical incision. In this cohort, this approach was associated with comparable incision-to-delivery times and no instances of conversion between incision types. Optimal management of operative obstetric trauma requires a multidisciplinary team and a thoughtful approach to incision selection.
